# Cardiac Function in Patients with Early Cirrhosis during Maximal Beta-Adrenergic Drive: A Dobutamine Stress Study

**DOI:** 10.1371/journal.pone.0109179

**Published:** 2014-10-03

**Authors:** Aleksander Krag, Flemming Bendtsen, Emilie Kristine Dahl, Andreas Kjær, Claus Leth Petersen, Søren Møller

**Affiliations:** 1 Department of Gastroenterology, Odense University Hospital, Odense, Denmark; 2 Gastro Unit, Medical Division, Hvidovre Hospital, University of Copenhagen, Copenhagen, Denmark; 3 Hvidovre Hospital, Department of Clinical Physiology Nuclear Medicine & PET, Rigshospitalet, Hvidovre Hospital, University of Copenhagen, Copenhagen, Denmark; 4 Centre of Functional Imaging and Research, Department of Clinical Physiology and Nuclear Medicine, Hvidovre Hospital, University of Copenhagen, Copenhagen, Denmark; Temple University, United States of America

## Abstract

**Background and aim:**

Cardiac dysfunction in patients with early cirrhosis is debated. We investigated potential cardiac dysfunction by assessing left ventricular systolic performance during a dobutamine stress test in patients with early cirrhosis.

**Patients and methods:**

Nineteen patients with Child A and B cirrhosis (9 with non-alcoholic cirrhosis) and 7 matched controls were included. We used cardiac magnetic resonance imaging to assess left ventricular volumes and cardiac output (CO) at rest and during maximal heart rate induced by increasing dosages of dobutamine and atropine.

**Results:**

Patients with cirrhosis and controls had an equal stress response, the heart rate and ejection fraction increased similarly and maximal heart rate was reached in all. At rest CO was higher in Child B patients than controls. During maximal stress, Child B patients had higher CO (10.6±2.7 vs. 8.0±1.8 L/min), left ventricle end diastolic volume (90±25 vs. 67±16 mL), left ventricular end diastolic volume (10±4 vs. 6±2 mL) and stroke volume (80±23 vs. 61±15 mL) than Child A patients. The systemic vascular resistance was lower in Child B than Child A patients (670±279 vs. 911±274 dyne*s*cm^−5^). The left ventricle mass increased by 5.6 gram per model for end stage liver disease (MELD) point. MELD score correlated with the end diastolic and systolic volume, CO, and stroke volume at rest and at stress (all p<0.05).

**Conclusion:**

In patients with early cirrhosis the chronotropoic and inotropic response to pharmacological stress induced by dobutamine is normal. With progression of the disease, the mass of the heart increases along with increase in cardiac volumes.

## Introduction

Several cardiac abnormalities have been described in cirrhosis. Diastolic and systolic dysfunction has been revealed in advanced cirrhosis and both seem to predict a poor outcome. Diastolic dysfunction is associated with reduced ascites clearance and increased mortality post transjungular intrahepatic portosystemic shunt (TIPS) operation [2]. Systolic dysfunction is associated with risk of developing hepatorenal syndrome [2]–[5]. Therefore a preserved systolic capacity with a high cardiac output at this stage of cirrhosis is related to better survival [5]. Furthermore, among patients who develop spontaneous bacterial peritonitis, a low cardiac output (CO) is associated with the development of hepatorenal syndrome and poor survival [6]. Several factors are found to differentiate patients with cirrhosis from healthy subjects. First, in advanced cirrhosis there are high levels of circulating noradrenaline and both beta-1 and -2 adrenoreceptors are down regulated. Beta-1 and -2 adrenoreceptors regulate the rate and the contractility of the heart [7], [8]. Second, the adrenergic positive inotropic effect on the heart is reduced in experimental cirrhosis [9]. Third, impaired chronotropic response to stress is a predictor of low survival in healthy individuals and in sepsis [10]. A blunted chronotropic response is found in previous studies in cirrhosis both during exercise, tilting, paracentesis, and infections [6], [11]–[13]. There is an increasing amount of data supporting a cardiac dysfunction in advanced cirrhosis with an impact on the risk of complications and survival [14]. However, cardiac systolic function in early cirrhosis has only been sparsely investigated. A cardiac dysfunction may be either subclinical, compensated at rest or in stable conditions and may only become clinically significant during circulatory stress [15]. Investigation of cardiac function by cardiac magnetic resonance imaging (CMRI) during maximal dobutamine stress is considered the gold standard for assessment of systolic abnormalities. We therefore aimed with CMRI to investigate left ventricular systolic performance at rest and during pharmacologically induced maximal beta-adrenergic drive in patients with mild cirrhosis.

## Material and Methods

Nineteen patients with Child A and B cirrhosis and 7 matched controls participated. Nine out of 19 patients had non-alcoholic cirrhosis. Patients were included if they had mild cirrhosis Child A or B without known heart disease. Patients were excluded if they had experienced gastrointestinal bleeding within the month preceding the study, showed signs of insulin-dependent diabetes, acute or chronic intrinsic renal or cardiovascular disease, abnormal electrocardiogram (ECG) (apart from prolonged QT intervals), or any acute medical conditions, such as infections, acute heart or lung diseases. Furthermore, alcohol abstinence for two months was required. To make sure none of the patients had an undiscovered heart disease, all patients had a single photon emission computed tomography (SPECT) myocardial perfusion scan done the week before the study. A normal SPECT is associated with a good prognosis regarding cardiac events and excludes ischemic heart diseases with high accuracy [16]. A negative history of arterial hypertension, cardiac and pulmonary diseases together with a normal clinical examination, apart from signs of portal hypertension and cirrhosis, was required. Except from beta-blockers, none of the patients were receiving any drugs that could interfere with the cardiovascular or renal function, thus no patients had indications for cardiovascular medications. A normal serum creatinine, together with normal fasting blood glucose was also required. All patients were screened for diabetes before inclusion. Among patients 11 were smokers and 4 in the control group smoked. If the patient or control was a smoker, extra care during clinical examination was taken to be sure they did not have a chronic lung disease or signs of arteriosclerosis.

Treatment with diuretics or beta-blockers was temporarily discontinued for 7 days before the investigations to eliminate a pharmacological influence on cardiac work or volume status. In total, 5 patients were treated with furosemide 40–80 mg/day, 9 patients were treated with spironolactone 100–400 mg/day and 6 patients were treated with propranolol 80–160 mg/day. Nine patients did not receive any medication for their cirrhosis. Water overload may induce subtle cardiac dysfunction and therefore all were put on a sodium-restricted diet of 80 mmol/day in the last 7 days before the investigations. A dietician instructed them written and verbally about sodium restriction.

### Stress testing

Dobutamine infusion started at 5 µg/Kg/min and was increased by 5 µg/Kg/min every third minute until a maximum of 40 µg/Kg/min or achievement of the pre-calculated age-adjusted maximal heart rate. The aim was to reach 85% of maximal heart rate defined as (220 – age) * 85%. If target heart rate was not achieved during maximal dobutamine infusion (40 µg/Kg/min), then atropine was added in refract doses of 0.25 mg. After reaching target maximal heart rate, infusion of dobutamine and atropine was continued throughout the CMRI. The total workload of the heart, as expressed by heart rate times blood pressure product, was calculated from the maximal systolic blood pressure achieved during maximal stress times maximal heart rate.

### Cardiac magnetic resonance imaging (CMRI)

The recordings were performed at Frederiksberg Hospital, University of Copenhagen in accordance with a standard protocol for CMRI as described in detail previously [17]. Briefly, CMRI was achieved with a 1.5 Tesla whole body scanner (Intera; Philips Medical Systems, MN, USA) using a dedicated hased array cardiac coil (Synergy; Philips Medical Systems, MN, USA). Following localisation of the long axis of the heart, continuous true short-axis slices were acquired using breath-hold ECG-triggered cine MRI gated prospectively. We applied 10 to 15 slices with 10 mm apart to measure the dimensions of the heart. The endocardial contours were drawn at the end-diastolic and end-systolic frame in all slices using standard software (Philips View Forum, release 3.2). End-diastolic volume (EDV) and end-systolic volume (ESV) were calculated by adding volume measurement in the end-diastole and end-systole, respectively from all slices. Based on EDV, ESV and heart rate LV stroke volume [1], ejection fraction (EF) and CO were calculated. For measurements of LV myocardial volume, epicardial contours were drawn at the end-diastolic frame in all slices. Myocardial mass was hereafter calculated by adding the differences between epi- and endocardial volumes with correction for the density of cardiac tissue (1.04 g/cm^3^). Cardiac index (CI) was calculated as CO divided by the total body surface area [18]. Arterial compliance [19] was assessed as SV/puls-pressure (systolic minus diastolic blood pressure) and the systemic vascular resistance (SVR) as MAP*80/CO.

### Hormones

A commercial available ELISA kit (Biomedica Gruppe, Vienna, Austria) was used to measure pro-atrial natriuretic peptide, ANP. Intra-assay and inter-assay coefficients of variation was 2% and 0.034 nmol/ml. An automated two-site sandwich immunoassay technique using chemilumin essence (ADVIA Centaur, Siemens, Germany) was used to measure BNP. The assay measures the physiologically active COOH-terminal peptide (77–108). The sensitivity of the assay was 2 pg/ml, intra-assay coefficient of variation was 1.2%, and inter-assay variation was 2.3%. Plasma renin concentration was determined by a commercially available two-site immunoradiometric assay (DGR International Inc.,USA). Samples were collected in ice-chilled test tubes containing aprotinin-heparin and EDTA.

The Regional Ethics Committee for Copenhagen and Frederiksberg municipalities approved the study (journal number KF 01-270675) and it is registered on clinicaltrials.gov (NCT00250315). All patients gave their written informed consent to participate in accordance with the Helsinki II declaration.

Statistical analyses were performed by unpaired Student's *t* test or the Mann-Whitney test and paired Student's *t* test or the Wilcoxon test, as appropriate. All reported P-values are two-tailed with values less than 0.05 considered significant. Correlations were performed with Persons regression analyses. The SPSS 20 statistical package (SPSS Inc., Chicago) was applied throughout.

## Results

Twenty patients were included in the study of which one was excluded due to hemochromatosis. The etiology of cirrhosis was alcohol in 10 patients, autoimmune in six patients, HCV in two patients and one had primary biliary cirrhosis. Demographic, clinical and biochemical characteristics of the participants are presented in table 1.

**Table 1:** Demographic, clinical, and biochemical characteristics of patients with cirrhosis and controls.

	Child A group (n = 12)	Child B group (n = 7)	Controls (n = 7)
Age (yr)	54±11	57±10	53±5
Gender (M/F)	4/8	5/2	2/5
Child-Pugh score	5.3±0.5	7.5±0.8	-
MELD score	6.3±4.0	10.3±2.8	-
BMI (kg/m^2^)	24±7	25±4	26±3
Aetiology (alcohol/no alcohol)	4/8	6/1	-
Diuretics (yes/no)	3/9	7/0	-
Beta-blocker treatment*	25%	43%	-
S-Sodium (135–145 mM)	142±4	139±5	-
S-Bilirubin (2–17 µmol/L)	13±9	15±5	-
S-Creatinine (49–121 µmol/L)	71±7	89±30	-
S-Albumin (37–48 g/L)	42±5	34±6	-
INR (0.9–1.2)	1.1±0.2	1.4±0.2	-
Pro-ANP nmol/L	2.9±1.4	3.6±1.2¤	2.1±0.3
BNP pg/mL	31.0±23	32.7±23.8	21.3±12.5
Renin ng/L	11 (6–19)	32 (18–98)¤	6 (4–9)

Mean ± SD, median and interquartile ranges. Reference interval in parentheses.

*The percentage of patients treated with beta-blockers before start of study. Compared with controls ¤ p<0.05.

Abbreviations:

Body Mass Index (BMI), International Normalized Ratio (INR), Model for End-stage Liver Disease (MELD).

### Baseline differences

**Figure 1:**
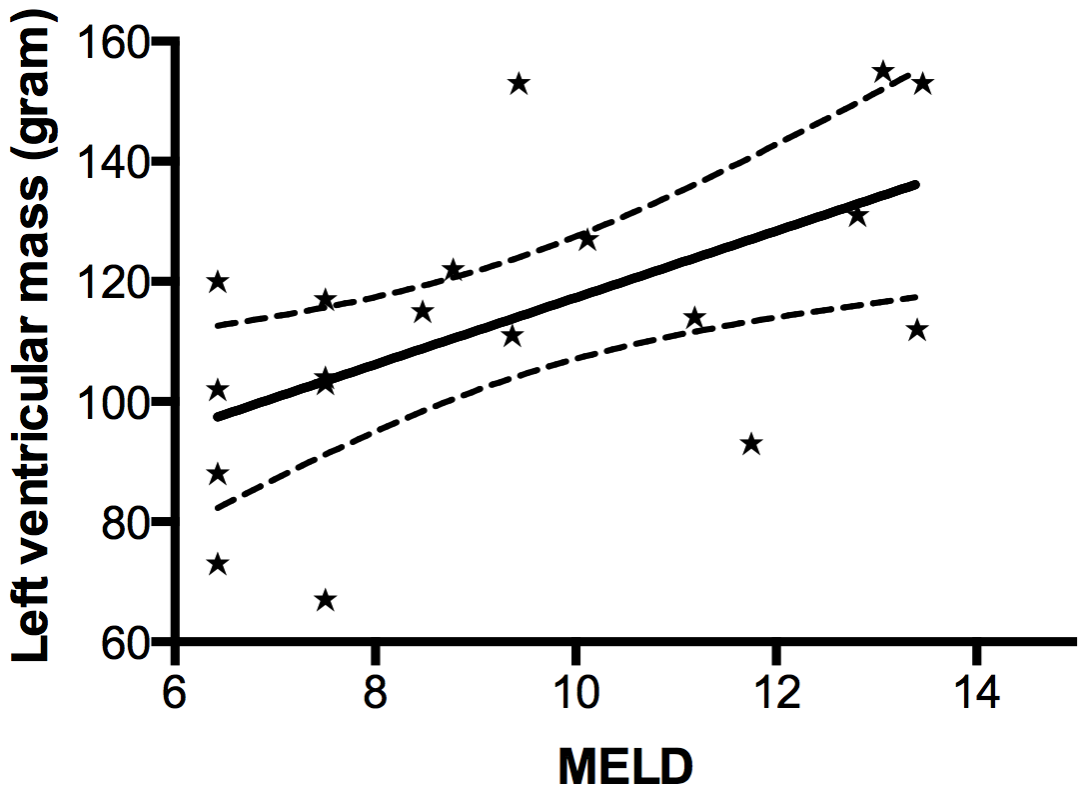
MELD score and left ventricular mass. The figure shows the correlation between Model for End-stage Liver Disease (MELD) score and the left ventricle mass at rest. For each MELD point the left ventricle mass increases with 5.6 gram, p<0.01. The two outer lines indicate a 95% confidence interval.

At rest Child B patients had higher CO compared to Child A patients (6.9±1.4 vs. 5.0±1.3 L/min, p = 0.009); similarly CI differed (3.7±0.8 vs. 2.8±0.6 L/min/m^2^, p = 0.02). The left ventricular mass correlated with MELD score (r = 0.58, p<0.01) and for each MELD point the left ventricle mass increased by 5.6 gram (Figure 1). At rest, the following volumes correlated positively with the MELD score and increased in volume for every MELD point: EDV (r = 0.70, p<0.01) ESV (r = 0.46, p<0.05), CO (r = 0.66, p<0.01) and SV (r = 0.69, p<0.01).

### Stress testing

**Figure 2:**
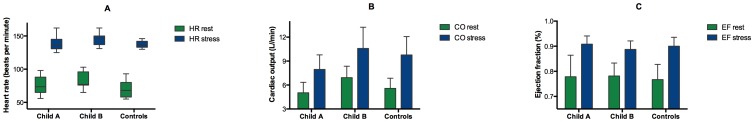
Response to dobutamine stress test. The figures show the response regarding heart rate (A), cardiac output (B) and ejection fraction (C) from rest to stress during maximal dobutamine infusion in Child A patients, Child B patients and controls. The bars show 95% confidence interval. Figure 2a: all groups show a similar significant increase in heart rate. Figure 2b: all groups show a similar significant increase in cardiac output. Cardiac output was significantly higher at rest in Child B patients compared to Child A patients, p<0.01. Figure 2c: all groups show a similar significant increase in ejection fraction.

The pre-calculated maximal heart rate was reached in all patients and the heart rate increased by 80% (62±16 beats/min) and 92% (66±15 beats/min) in cirrhosis and controls, NS (Figure 2a, Table 2). The cardiac work, as reflected by the heart rate blood pressure product, increased by 84% (8854±3915 mmHg/min) in Child A, 87% (9232±2418 mmHg/min) in Child B and 100% (9979±4857 mmHg/min) in controls, NS (Table 2). The dose of atropine (0.25 mg per dose) did not differ between cirrhosis and controls, p = 0.94 (0.26±0.7 vs. 0.29±0.5 dose of atropine). There was no difference in the increase in heart rate and heart rate blood pressure product between alcoholic and non-alcoholic cirrhosis. In all three groups, there were a significant increase from rest to stress in CO (mean difference 2.9±1.4, 3.7±1.8 and 4.2±1.9 L/min, all p<0.001) (Figure 2b), CI (mean difference 1.7±0.8, 1.9±0.9 and 2.2±1.0 L/min/m^2^, all p<0.001) and EF (mean difference 13±6, 11±5 and 13±5%, all p<0.01) (Figure 2c) in Child A, Child B and controls, respectively. Likewise, there was a significant decrease in EDV (mean difference 21.3±16.5 mL p = 0.001, 17.1±12.8 mL p = 0.012 and 20.4±11.2 mL p = 0.003), ESV (mean difference 14.8±11.7 mL p = 0.001, 13.3±7.4 mL p = 0.003 and 16.4±7.1 mL p = 0.001) and SVR (mean difference 822±645 dyne*s*cm^−5^ p = 0.001, 438±150 dyne*s*cm^−5^ p<0.001 and 664±251 p<0.001 dyne*s*cm^−5^) during maximal stress, in Child A, Child B and controls, respectively (Table 2). SV decreased significantly in Child A patients from rest to stress (by 6.5±10.1 mL, p = 0.047) but not in Child B patients (3.9±13.3 mL, p = 0.47) or controls (4.0±9.8 mL, p = 0.32). AC decreased in Child A (by 0.4±0.4 mL/mmHg, p = 0.006) and Child B (by 0.7±0.7 mL/mmHg, p = 0.035) but not in controls (by 0.5±0.8 mL/mmHg, p = 0.15). There was no difference in mean arterial pressure (MAP) from rest to stress in any of the three groups.

**Table 2:** Cardiovascular function in 19 patients with cirrhosis and 7 controls at rest and after pharmacological stress with dobutamine +/− atropine.

	Child A group (n = 12)		Child B group (n = 7)		Controls (n = 7)	
	Rest	Stress	Rest	Stress	Rest	Stress
HR min^−1^	76±13	138±10*	83±13	145±10*	71±14	137±5*
MAP mmHg	100±17	91±15	93±11	93±14	98±22	96±11
CO L/min	5.0±1.3	8.0±1.8*	6.9±1.4**∞**	10.6±2.7∞*	5.6±1.3	9.8±2.3*
CI L/min*m^2^	2.9±0.6	4.5±0.9*	3.7±0.8**∞**	5.6±1.4*	2.9±0.9	5.2±1.5*
SV mL	68±16	61±15¤	84±22	80±23**∞**	80±13	76±17
EF %	78±9	91±3*	78±5	89±4*	77±6	90±4*
LV EDV ml	89±26	67±16*	107±27	90±25**∞¤**	105±16	86±17*
LV ESV ml	21±13	6±2*	23±8	10±4∞*	25±7	8±3∞*
LV mass g	108± 20		123±30		125±30	
AC mL/mmHg	1.2±0.5	0.9±0.2*	1.8±0.8	1.1±0.2**∞¤**	1.5±0.6	1.1±0.3
SVR dyne*s*cm^−5^	1733±857	911±274*	1138±267	670±279*	1428±349	763±191*

Mean ± SD.

Comparison between rest and stress: ¤ p<0.05.

Comparison between rest and stress: * p<0.01.

Comparison with Child A patients: ∞ p<0.05.

Abbreviations:

Arterial Compliance (AC), Cardiac Index (CI), Cardiac Output (CO), Ejection Fraction (EF), Heart Rate (HR), Left Ventricle (LV), End-Diastolic Volume (EDV), End-Systolic Volume (ESV), Mean Arterial blood Pressunre (MAP), Stroke Volume (SV), Systemic Vascular Resistance (SVR).

### Stress differences

At stress CO, SV, EDV, ESV and AC was significantly higher in Child B patients compared to Child A patients (all p<0.05), but EF and SVR did not differ. There were no differences in any volumes when comparing Child A and controls or Child B and controls.

**Figure 3:**
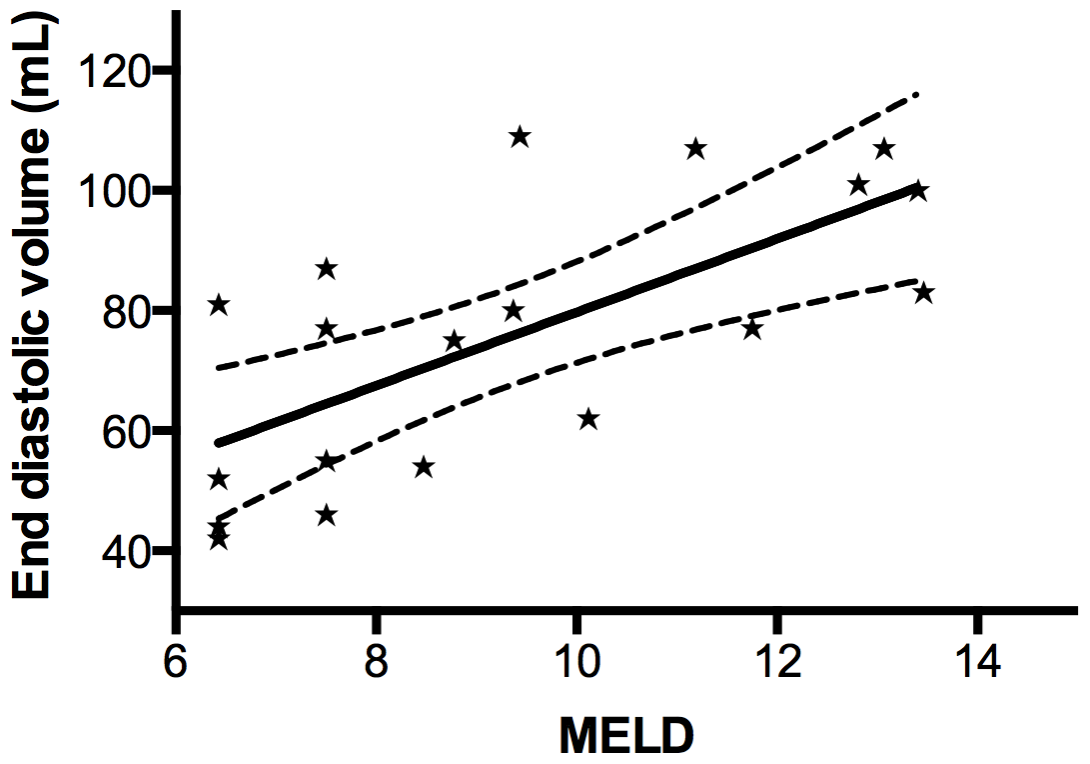
MELD score and end diastolic volume. The figure shows the correlation between model for end-stage liver disease (MELD) score and end diastolic volume during maximal stress, p = 0.001. The two outer lines indicate a 95% confidence interval.

As seen at baseline, EDV, ESV, SV, and CO correlated significant positively with the MELD score during maximal stress (EDV (r = 0.69, p<0.01), ESV (r = 0.56, p<0.01), SV (r = 0.68, p<0.01) and CO (r = 0.59, p<0.01)) (Figure 3). There was no difference in the response to stress between alcoholic and non-alcoholic cirrhotic patients.

### Hormones

At rest, pro-ANP was higher in Child B patients compared to controls (3.6±1.2 vs. 2.1±0.3 nmol/L, p<0.01), Table 1. Both Child A and Child B patients had higher pro-ANP levels during maximal stress than controls (p<0.05 and p<0.01) but only in Child B patients pro-ANP significantly increased from rest to stress by 21% (0.77±0.38 nmol/L, p<0.05). BNP remained unchanged in both groups during stress. At rest, renin was slightly higher in cirrhosis compared to controls, most pronounced in Child B patients (p<0.05). During dobutamine induced circulatory stress, renin increased similar in all groups (Child A (p<0.01, by 22.5 ng/L (11–75)), Child B (p<0.02, by 29 ng/L (6–200)), controls (p<0.02, by 11 ng/L (5–45)).

## Discussion

The definition and consequences of cirrhotic cardiomyopathy is still debated [14], [20]. Despite a considerable amount of research in this area many questions still remain unanswered. Most studies investigating cirrhotic cardiomyopathy have mainly been performed with various stress tests or echocardiography. The present study is the first to assess systolic cardiac function by dobutamine stress CMRI in early cirrhosis. Our main findings are: 1) A sufficient increase in heart rate suggesting intact beta-adrenergic response in early cirrhosis. 2) The increase in the blood pressure-heart rate product in Child A and Child B patients during dobutamine infusion was similar to controls. Thus, the chronotropic and inotropic response to dobutamine during stable conditions seems intact in early cirrhosis. 3) With progression of the disease the mass of the heart increases along with an increase in cardiac volumes.

In an experimental study on cirrhotic rats a desensitization of myocardial beta-adrenoreceptors was found [7]. The rats where stimulated with isoprenaline and needed a significantly higher dose than controls before reaching target heart rate. Analyses showed a down-regulation of the density of beta-1-adrenoreceptors in the myocardial cells. We would therefore have expected a higher dose of dobutamine required to reach maximal heart rate in cirrhosis than controls. This difference is likely due to down regulation of beta-1-adrenoreceptors, which may not appear until advanced stages of cirrhosis where the sympathetic nervous system is highly activated [4]–[6], [21]. However, it should be emphasized that heart rate is a surrogate readout of beta-adrenergic receptor responsiveness and does not provide mehanistic explanations such as downregulation or desensitization of receptors. However, it is easy to measure in a clinical setting and relate directly to cardiac output.

It has been proposed that stress could unmask cirrhotic cardiomyopathy. Two studies examined cirrhotic patients with bicycle ergometry and both found an inability to reach maximal heart rate with a lower total cardiac work [12], [22], [23]. Grose et al. found unaltered EF and an increase in EDV in patients with cirrhosis [12] and Wong et al. found a prolonged isovolumic relaxtion time, increased left ventricle wall thickening and less oxygen consumption in patients with cirrhosis [22]. Both studies suggest decreased diastolic and systolic functions as signs of cirrhotic cardiomyopathy. In accordance with this, Kim et al. found by stress echocardiography 25% of the patients to have blunted left ventricle response during maximal dobutamine infusion [24]. It has been questioned whether these bicycle or other exercise induced stress test indicate cirrhotic cardiomyopathy. Lemyze et al. suggest that the inability to reach maximal heart rate and thereby less produced work in bicycle ergometry tests may be explained by deconditioning, malnutrition-associated muscle weakness or anemia [25]. In support of this, studies have found that during exercise cirrhotic patients have decreased glucose uptake in the skeletal muscles, lower endogen glucose production together with a shift in oxidation from glucose towards lipids in energy production [26]–[28]. Furthermore, pulmonary dysfunction is common in cirrhosis [29] and may contribute to the insufficient amount of work produced during exercise tests [30].

In normal physiologic circumstances the systolic response at stress may be different from what is observed by dobutamine infusion. Sepsis, hypovolaemia, or physical work results in differences in loading conditions. Thus the venous return and preload increase with physical work, whereas preload is low in advanced stages with central hypovolemia [31], [32]. Our patients showed a normal response towards stress, but in the setting of a decreased afterload as reflected by the significant fall in SVR. Likewise, MAP remained unchanged during the whole study despite a steep increase in CO and heart rate. Dobutamine has a strong affinity for beta-1-adrenoreceptors and results in an increased heart rate, contractility and enhanced AV node conduction. But dobutamine also has affinity for beta-2-adrenoreceptors and induces vasodilation [33]. This explains the observed decrease in SVR and afterload. In a previous study in patients with advanced cirrhosis we increased the afterload by terlipressin and observed a decrease in EF, CO and an increase in EDV. An increase in afterload requires an increase in total workforce, which was clearly impaired in these patients with advanced cirrhosis [34]. A number of factors related to the stage of cirrhosis may explain why systolic function as assessed in this study is adequate. Most patients with advanced cirrhosis have chronic elevation of proinflammatory cytokines, such as IL-6 and TNF-alfa and endotoxins, which represent a chronic low-grade inflammatory state [35]. The activation of cytokines, vasoactive hormones, and alteration in circulatory function in advanced cirrhosis and ascites without overt sepsis is similar to that seen in sepsis and septic shock without cirrhosis [35]. In septic shock it is estimated that approximately 40% of the patients develop myocardial dysfunction characterised by decreased systolic contractility and impaired diastolic relaxation [36], [37]. In the cardiodepressant state, adrenal insufficiency (particularly with acute stresses), may result in critical illness related corticosteroid insufficiency [15], [21]. In early stages of stable cirrhosis these factors are not in force. This study did not assess diastolic function. However, diastolic function has been investigated in a number of studies and seems related to clinical outcome and may be evident in all stages of disease including early stages. Two recent large studies assessed diastolic dysfunction in cirrhotic patients using echocardiography [24], [38], [39]. Nazar et al. found mild diastolic dysfunction frequent in cirrhotic patients and unrelated to circulatory dysfunction, ascites and HRS [38]. Ruiz-del-Arbol et al. did a one-year follow up study and found that the degree of left ventricular diastolic dysfunction correlated with survival rate and development of hepatorenal syndrome [39]. Furthermore, autonomic dysfunction may also play a role. In a previous study we found that after dobutamine stress the time to resume heart rate of 100 beats/min was longer in cirrhosis than in controls [40]. It is well known that patients with cirrhosis have altered hemodynamics leading to a hyperdynamic syndrome [14], [41]. We found a significant higher CO in Child B patients compared to Child A patients. Furthermore, left ventricle mass correlated with the Child-Pugh score and increased with 2.8 gram per MELD score. Most likely, the observed increase in left ventricle mass is a physiology response to the increased work as reflected by increased CO. The increased CO also explains why Child B patients showed a significant higher stroke volume, end systolic volume and end diastolic volume than Child A patients during stress. These are likely the earliest changes towards the hyperdynamic syndrome seen in advanced cirrhosis with decreased SVR, increased CO, increased left ventricular mass together with subtle hormonal changes [42]. Physiological adaptions to protect the circulation and organ perfusion against the thread from progressive vasodilation [43]. Thus at this stage cardiac function and compensatory reserve in terms of ability to increase CO is intact and cardiac function is increased rather than depressed.

The slight increase in renin, mainly seen in Child B patients, support that the patients were in early stages of cirrhosis with a beginning of hemodynamic derangement and activation of vasoactive hormones [42]. This fit well with the fact that Child B patients had an increased CO compared to controls. The increase in renin after dobutamine infusion was similar in cirrhosis and controls and is likely a direct effect through stimulation of the beta_1_ adrenergic receptors.

## Conclusions

In stable early cirrhosis with normal loading conditions the cardiac systolic response to pharmacological stress is normal. With progression of the disease the mass of the heart increases along with an increase in cardiac volumes. Future follow-up studies should look for changes in patients with advanced cirrhosis and the long-term impact of cardiac dysfunction in the development of complications of cirrhosis.
